# Human Infant Memory B Cell and CD4+ T Cell Responses to HibMenCY-TT Glyco-Conjugate Vaccine

**DOI:** 10.1371/journal.pone.0133126

**Published:** 2015-07-20

**Authors:** Angela Fuery, Peter C. Richmond, Andrew J. Currie

**Affiliations:** 1 School of Paediatrics and Child Health, The University of Western Australia, 35 Stirling Highway, Perth, WA 6009, Australia; 2 Wesfarmers Centre for Vaccines and Infectious Diseases, Telethon Kids Institute, The University of Western Australia, 100 Roberts Road, Perth, WA 6008, Australia; 3 Princess Margaret Hospital for Children, Roberts Road, Perth, WA 6008, Australia; 4 School of Veterinary & Life Sciences, Murdoch University, Murdoch, WA 6150, Australia; Public Health England, UNITED KINGDOM

## Abstract

Carrier-specific T cell and polysaccharide-specific B cell memory responses are not well characterised in infants following glyco-conjugate vaccination. We aimed to determine if the number of Meningococcal (Men) C- and Y- specific memory B cells and; number and quality of Tetanus Toxoid (TT) carrier-specific memory CD4+ T cells are associated with polysaccharide-specific IgG post HibMenCY-TT vaccination. Healthy infants received HibMenCY-TT vaccine at 2, 4 and 6 months with a booster at 12 months. Peripheral blood mononuclear cells were isolated and polysaccharide-specific memory B cells enumerated using ELISpot. TT-specific memory CD4+ T cells were detected and phenotyped based on CD154 expression and intracellular TNF-α, IL-2 and IFN-γ expression following stimulation. Functional polysaccharide-specific IgG titres were measured using the serum bactericidal activity (SBA) assay. Polysaccharide-specific Men C- but not Men Y- specific memory B cell frequencies pre-boost (12 months) were significantly associated with post-boost (13 months) SBA titres. Regression analysis showed no association between memory B cell frequencies post-priming (at 6 or 7 months) and SBA at 12 months or 13 months. TT-specific CD4+ T cells were detected at frequencies between 0.001 and 0.112 as a percentage of CD3+ T cells, but their numbers were not associated with SBA titres. There were significant negative associations between SBA titres at M13 and cytokine expression at M7 and M12. *Conclusion*: Induction of persistent polysaccharide-specific memory B cells prior to boosting is an important determinant of secondary IgG responses in infants. However, polysaccharide-specific functional IgG responses appear to be independent of the number and quality of circulating carrier-specific CD4+ T cells after priming.

## Introduction

The introduction of glyco-conjugate vaccines to infant and toddler vaccination schedules has led to a reduction in invasive diseases such as meningitis and sepsis, which result from infection by polysaccharide-encapsulated bacteria [1, 2]. The conjugation of a carrier protein, such as Tetanus Toxoid (TT), to targeted bacterial polysaccharide antigens is a key structural feature of glyco-conjugate vaccines. This enables T-dependent anti-polysaccharide antibody responses to be induced in infants, overcoming their potential deficiencies in T-independent polysaccharide-specific responses. Despite the success of glyco-conjugate vaccines, there is evidence that protective IgG responses wane more rapidly in infants than toddlers following priming schedules [3], making the need to understand the nature of the cellular response to these vaccines in this age group imperative. In particular, little is known of the ideal B cell phenotype (i.e. memory and/or plasma cell) following a priming schedule in infants, and whether the number and quality of primed carrier protein-specific CD4+ T cells may influence the persistence of protective IgG.

A number of studies in animal models have identified that B cells primed at initial antigen encounter can become either memory B cells or plasma cells, the latter of which are terminally differentiated and short- or long- lived [4, 5]. In studies of glyco-conjugate vaccines, polysaccharide-specific plasma cells [6] and memory B cells [6–12] have been measured in the peripheral blood following priming and booster doses, and two studies in infants have identified that Men C-specific memory B cells present post-priming are good predictors of the persistence of Men C-specific IgG at 12 months prior to boosting [7, 11].

Despite knowledge gained from these studies, other studies of glyco-conjugate vaccine immunogenicity suggest that B cell kinetics are perhaps more strongly influenced by primed carrier protein-specific CD4+ T cells than initially thought. For example, differences in the rapidity of waning IgG have been observed between glyco-conjugates which differ in both their capsular components and carrier protein [13, 14], suggesting potential influence from carrier protein-specific CD4+ T cells. It remains unclear whether the magnitude of carrier protein-specific CD4+ T cell responses post-priming and pre-boost, affect the persistence of polysaccharide-specific IgG in infants.

We have studied vaccine-specific B cell and T cell responses in a cohort of infants vaccinated with a combined *Haemophilus influenzae* b (Hib), Men C and Y glyco-conjugate vaccine which uses Tetanus Toxoid (TT) as a carrier protein (HibMenCY-TT; MenHibrix, GSK Biologicals). This vaccine was recommended for use in children aged 6 weeks to 18 months who are at increased risk of Meningococcal infection in the US in 2012. Our investigations enumerated Men C- and Y- specific memory B cells and TT-specific CD4+ T cells, and measured the cytokine producing potential of TT-specific CD4+ T cells. We then established associations between these cellular measurements and persistent and post-booster functional IgG responses.

## Materials and Methods

### Study population

The study was conducted as an investigator-led add-on to a GlaxoSmithKline (GSK) sponsored phase II multi-centre, open, randomised and controlled study of the HibMenCY-TT vaccine, described elsewhere [15] and registered at www.clinicaltrials.gov (NCT00134719). Briefly, healthy infants between 6 and 12 weeks of age were randomised to receive HibMenCY-TT (GSK, Rixensart, Belgium), MenC-CRM_197_ (Pfizer, New York, USA) + Hib-TT (Sanofi Pasteur, Lyon, France) or Hib-TT (Sanofi Pasteur) in a 2-4-6 month priming schedule. A booster dose of HibMenCY-TT vaccine was given to all participants at 12–15 months. Blood sampling was conducted after either 2 or 3 doses of prime, and pre and post boosting for measurement of serum IgG concentrations and functional IgG through the serum bactericidal antibody (rSBA) assay using rabbit complement as the external complement source.

This add-on study of cellular responses (Princess Margaret Hospital Ethics approval number 1180/EP) was conducted at the Perth site. Parents/guardians of subjects gave written informed consent for collection of an additional 5–10mL blood sample into sterile tubes containing heparin sodium for injection (Pfizer, New York, USA), for isolation of peripheral blood mononuclear cells (PBMCs). In this study, we present data from infants in the first treatment group who received HibMenCY-TT vaccine in the priming schedule, and who also had samples available at each time point, a total of 44 infants (Table 1).

**Table 1:** Number of subjects vaccinated and studied at each time point.

	Time-point (months)					
	2	4	6	7	12	13
***HibMenCY-TT***	44	44	44		44	
***Blood sampling***			21	23	44	44

### Concomitant Vaccines

In addition to study vaccines, subjects were immunised according to the Australian vaccination schedule (http://www.nevdgp.org.au/info/immunisation/table_1_7_1.htm, 2003). All priming doses were therefore co-administered with combined diphtheria-tetanus-acellular pertussis-hepatitis B-inactivated Poliovirus vaccine (DTPa-HBV-IPV, Infanrix Penta, GSK, Rixensart, Belgium) and 7-valent pneumococcal conjugate vaccine (7vPCV; Prevenar, Pfizer, New York, USA). All booster doses were co-administered with Measles, Mumps and Rubella (MMR) vaccine (MMRII, Merck, Whitehouse Station, USA) and Varicella vaccine (Varivax, Merck).

### Men C and Men Y SBA responses

Men C- and Men Y- specific rSBA titre was measured, in the presence of rabbit complement, according to protocols used by GSK in the larger study cohort [15]. An arbitrary value of 1 was given to SBA values falling below the assay cut-off of 1/8.

### PBMC preparation

PBMCs were prepared according to methods described previously [16], prior to storage in liquid nitrogen.

### *In vitro* T cell cultures

Cryopreserved PBMCs were thawed rapidly in a water bath at 37°C, added to R-10 culture media, washed and resuspended in RPMI 1640 medium supplemented with 10% Heat Inactivated Foetal Calf Serum (HI-FCS; SAFC, Brooklyn, Australia), 2-mercaptoethanol (Sigma-Aldrich, Castle Hill, Australia), glutamax (Life Technologies, Melbourne, Australia), 4-(2-hydroxyethyl)-1-piperazineethanesulfonic acid (HEPES; Life Technologies), antibiotic-antimycotic (Life Technologies) and sodium pyruvate (Life Technologies) at a density of 2x10^6^ PBMCs/mL. PBMCs were plated in sterile polypropylene round-bottomed 96-well plates (Corning Costar, Lowell, USA) in a final volume of 250μL, with 5x10^5^ PBMCs per well. PBMCs were stimulated with 0.9% (w/v) NaCl (Baxter, Old Toongabbie, Australia) as a negative control, 10μg/mL TT (kindly provided by GSK, Rixensart, Belgium) or 2.5μg/mL Staphylococcal Enterotoxin B (SEB; Sigma-Aldrich) as a positive control. PBMCs were stimulated for 48 hours in a 37°C humidified incubator (Thermo Scientific, Waltham, USA) with 5% CO_2_, and 3μg/mL Brefeldin A solution (eBioscience, San Diego, USA) was added to wells for the final 16 hours of culture. Following culture, cells were harvested by gentle pipetting and washed prior to staining for expression of surface and intracellular markers.

### Flow Cytometry

TT-stimulated and control PBMCs were washed in polystyrene round-bottomed 96 well plates (Corning Costar, Lowell, USA) and stained for surface expression of CD3 (Pacific blue; UCHT1 clone, BD) and CD4 (APC-H7; RPA-T4 clone, BD) in the dark for 30 minutes at 4°C. After washing, cells were fixed with pre-diluted FACSLyse solution (BD Biosciences, San Jose, USA) for 10 minutes at room temperature (RT), followed by permeabilisation with pre-diluted Perm 2 solution (BD Biosciences) for 10 minutes at RT, according to the manufacturer’s instructions. Cells were then washed in FACS buffer consisting of phosphate buffered saline (PBS; Sigma-Aldrich), 2% (v/v) HI-FCS (SAFC), 2% (w/v) Bovine Serum Albumin (BSA; Sigma-Aldrich) and 0.01% (v/v) NaN_3_ (Sigma-Aldrich) prior to staining for expression of intracellular markers TNFα (FITC; MAb11 clone; eBioscience), CD154 (PE; TRAP-1 clone; BD), IL-13 (PerCPCy5.5; JES10-5A2 clone, BioLegend), IL-2 (APC; MQ1-17H12 clone, BioLegend), and IFN-γ (PE-Cy7; B27 clone, BD) for 30 minutes in the dark at 4°C. Cells were washed and re-suspended in pre-diluted stabilising fixative (BD Biosciences) prior to analysis on a BD FACS Canto II flow cytometer. Voltages were set on FACSDiva software (BD Biosciences), and these remained unchanged throughout acquisition of data from the entire cohort. Following doublet exclusion a minimum of 100,000 lymphocyte events were collected. Fluorescence Minus One (FMO) controls were included in all experiments. Data was analysed on FlowJo software (Treestar, Ashland, USA). Compensation settings were applied following data acquisition using FlowJo software. To determine a positive signal for all CD154 staining, the signal from un-stimulated samples was subtracted from TT-stimulated samples to determine a TT-specific ΔCD4+CD154+ signal. Group allocation was blinded at the time of cell culture, data acquisition and analysis.

### Memory B cell culture

Methods were adapted from a method previously described for Pneumococcal antigens [17]. Briefly, thawed PBMCs were re-suspended in RPMI 1640 medium supplemented with 10% HI-FCS (SAFC), 2-mercaptoethanol (Sigma-Aldrich), glutamax (Life Technologies), HEPES (Life Technologies), antibiotic-antimycotic (Life Technologies) and sodium pyruvate (Life Technologies) at a density of 1x10^6^/mL. Two-hundred micro-litre aliquots were plated onto polypropylene round-bottomed 96-well culture plates (Corning Costar). Thirty micro-litres of stimulation mix comprising; CpG-2006 ODN (TIB MOLBIOL, Berlin, Germany), recombinant IL-2 (Prospec-Tany, Ness-Ziona, Israel) and recombinant IL-15 (Prospec-Tany) was added to each well to give a final concentration of 3μg/mL, 10ng/mL and 10ng/mL respectively. Cells were stimulated for 6 days in a 37°C humidified incubator (Thermo Scientific) with 5% CO_2_ prior to commencement of ELISpot.

### Preparation of ELISpot plates

96 well polyvinylidene difluoride (PVDF) membrane ELISpot plates (Merck Millipore, Billeria, USA) were coated with 5μg/mL Meningococcal C polysaccharide (NIBSC, Potters Bar, UK), 5μg/mL Meningococcal Y polysaccharide (NIBSC) or 10 μg/mL Hib PRP polysaccharide (NIBSC) conjugated to methylated human serum albumin (mHSA; NIBSC) at an equivalent dose, or 10μg/mL TT (GSK, Rixensart, Belgium) in sterile PBS. Positive control wells were coated with 10μg/mL goat anti-human Ig (Life Technologies, Mulgrave, Australia) in sterile PBS. Negative control wells were coated with sterile PBS. Plates were stored sealed at 4°C for up to 6 days when required. Prior to the addition of cells, plates were washed 3 times in sterile PBS and blocked in RPMI 1640 medium supplemented with 10% HI-FCS, 2-mercaptoethanol, glutamax, HEPES, antibiotic-antimycotic and sodium pyruvate at 37°C for 30–120 minutes.

### Detection of memory B cells

CpG/IL-2/IL-15-stimulated PBMCs were washed 3 times in ELISpot cell wash buffer containing PBS (Sigma-Aldrich), 0.744g/L EDTA (Sigma-Aldrich) and 0.5% HI-FCS (SAFC) prior to re-suspension in R-10 culture medium at a density of 2x10^6^ cells/mL. One hundred micro-litres of cell suspension was added to each well of the pre-coated ELISpot plate. To enable spot separation in positive control wells, cells were added at densities of 2x10^2^ and 2x10^3^ cells/well. Plates were incubated in a 37°C humidified incubator with 5% CO_2_ overnight. Cells were then discarded and the plates washed 4x in PBS containing 0.25% (v/v) Tween (TW) followed by 1x with PBS. Bound IgG antibodies were detected using a 1/5000 dilution of goat anti-human IgG γ chain-specific alkaline phosphatase (AP) conjugate (Calbiochem, Darmstadt, Germany) in R-10 culture media at RT for 4 hours. Plates were then washed 4x with PBS/0.25% (v/v) TW followed by 3x with H_2_O. An AP conjugate substrate kit (Bio-Rad, Hercules, CA, USA) was used for detection of antibody forming cells (AFCs), prior to rinsing with water, drying in a drying oven (Memmert, Schwabach, Germany) at 60°C and counting. AFCs were counted on an AID ELISpot reader (Autoimmun Diagnostika, Strassberg, Germany) using AID software version 2.9I. Settings were identical for all plates and spots below an intensity of 13 arbitrary units were not counted and considered background.

### Statistical methods

Non-parametrically distributed ELISpot data and TT-specific CD4+ T cell data was statistically analysed using independent samples Mann-Whitney U-tests when comparing between 2 priming doses (M6) and 3 priming doses (M7), whilst all paired samples (all other time points) were compared using related samples Wilcoxon signed rank tests. When analysing ELISpot data, 5 memory B cells (spots) per sample were considered to be the minimum detection limit of the assay for all antigens. The association between non-parametric memory B cell values and CD4+CD154+ T cell frequencies and log-transformed SBA values were assessed using linear regression. Differences and associations were considered significant if the p value was less than 0.05. We have chosen to describe all statistics carried out and have not made adjustments for multiple comparisons, as these were exploratory analyses. Statistical analyses were performed by using the statistical packages SPSS 20.0 (SPSS Inc., Chicago, USA) and Prism (GraphPad Software Inc., La Jolla, USA).

## Results

### Population characteristics

Forty-four subjects (average age at enrolment was 9.03 weeks; 24 male) had PBMCs available at all time points following priming and pre- and post- boosting (Table 1).

### Men C- and Men Y- specific SBA responses

**Fig 1:**
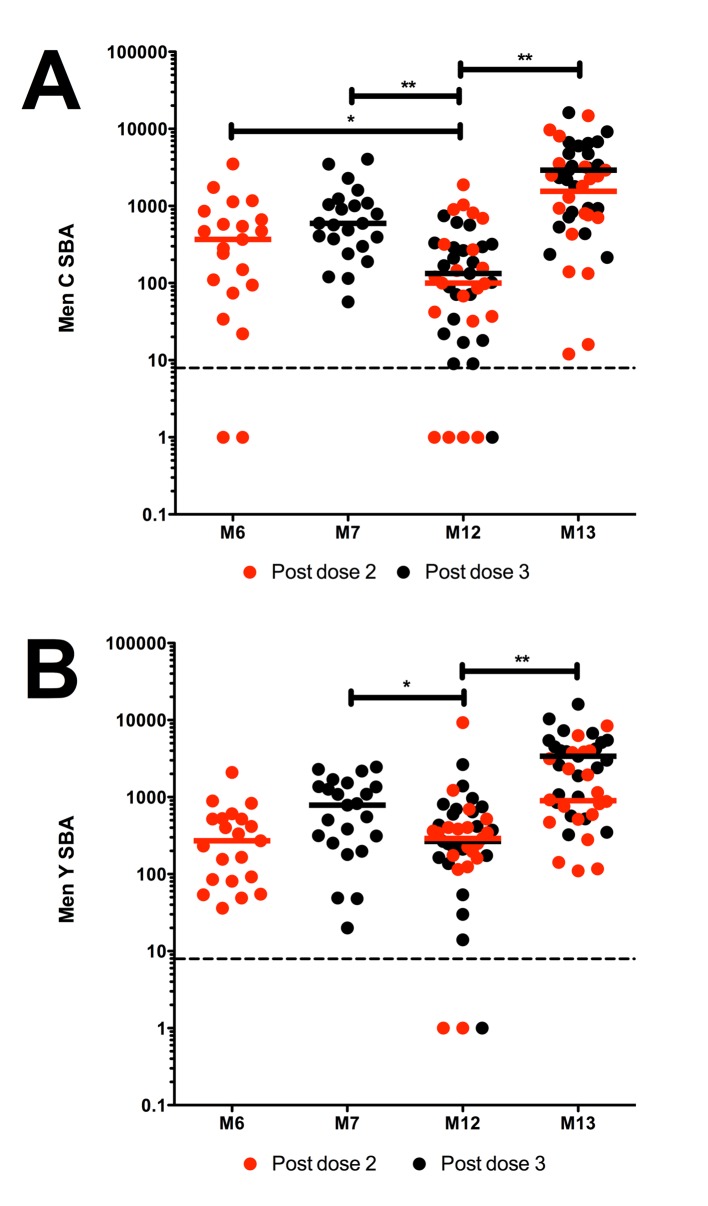
Men C- and Men Y- specific SBA titres following HibMen CY-TT vaccine. **A.** Men C- and **B.** Men Y- specific SBA titre after 2 (M6) or 3 (M7) priming doses, and prior to (M12) and 1 month post (M13) the booster dose of HibMenCY-TT vaccine. Red dots represent paired samples from individuals who had blood taken following 2 doses of HibMenCY-TT prime, whilst black dots represent those samples from individuals who had blood taken after 3 doses of HibMenCY-TT prime. Horizontal bars indicate median values. The dashed line represents the assay limit of detection. p(**)≤0.01 and p(*)≤0.05 indicating significance of independent samples Mann-Whitney U tests or related samples Wilcoxon signed rank tests.

SBA responses were representative of the larger cohort studied [15], reflecting a typical infant response to meningococcal conjugate vaccine with a good primary response, rapid waning and an anamnestic booster SBA response (Fig 1). Prior to boosting at month (M) 12, there was a significant decrease (**p<0.001 and *p = 0.026 respectively) in Men C- and Men Y- specific SBA titres from those at M7 (Fig 1). Compared to M6, Men C-specific SBA titres at M12 were significantly reduced (*p = 0.027) but the difference was not significant for Men Y. A booster dose of HibMenCY-TT significantly increased both Men C- and Men Y- specific SBA titres (**p<0.001 for both), with all subjects achieving titres above the protective threshold at M13.

### Men C- and Men Y- specific memory B cell responses

**Fig 2:**
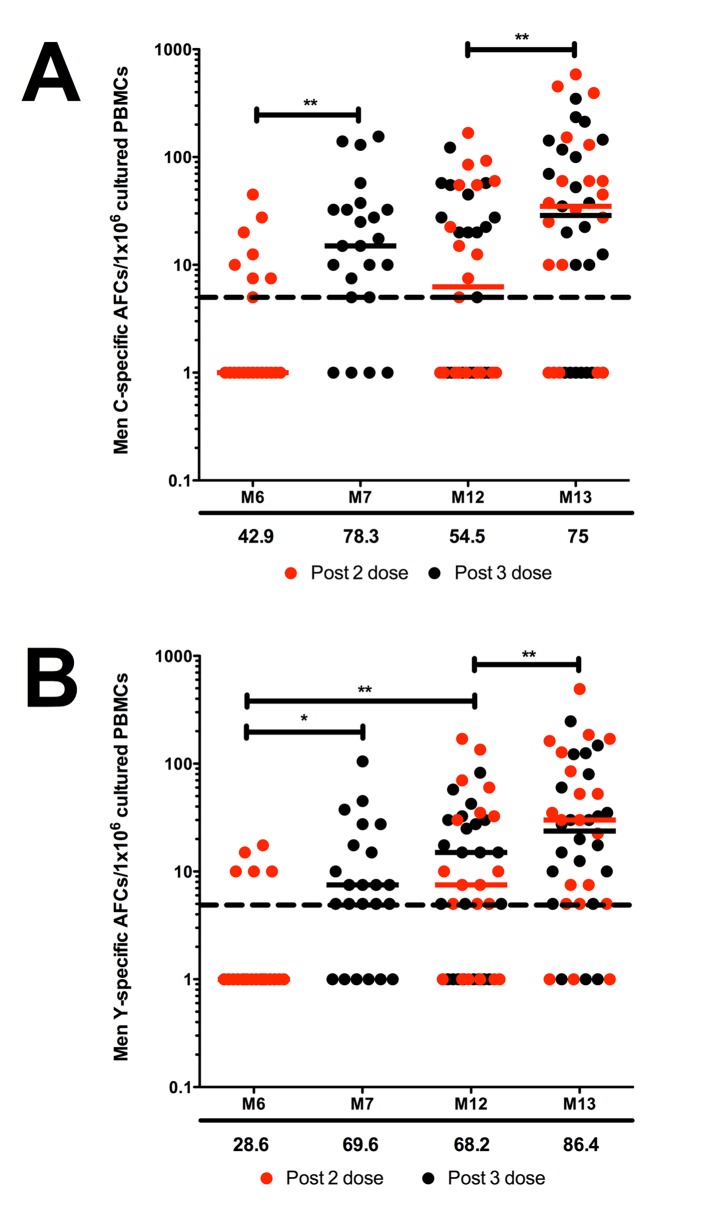
Men C- and Men Y- specific memory B cell responses following HibMenCY-TT vaccine. **A.** Men C- and **B.** Men Y- specific Antibody Forming Cells (AFCs) per 1x10^6^ cultured PBMCs after 2 (M6) or 3 (M7) priming doses, and prior to (M12) and 1 month post (M13) the booster dose of HibMenCY-TT, where each point represents the response from one individual and horizontal bars indicate median values. Individuals with no detectable spots were given an arbitrary value of 1. Red dots represent paired samples from individuals who had blood taken following 2 doses of HibMenCY-TT prime, whilst black dots represent those samples from individuals who had blood taken after 3 doses of HibMenCY-TT prime. The dashed line represents the assay limit of detection. The numbers below the x axis of each graph indicate the percentage of subjects with detectable memory B cells specific for that antigen at that time point. p(**)≤0.01 and p(*)≤0.05 indicating significance of independent samples Mann-Whitney U tests or related samples Wilcoxon signed rank tests.

We detected a typical conjugate vaccine profile of memory B cell induction, with an increase in the number of Men C- and Men Y- specific memory B cells after each priming dose where there were significantly more Men C- and Men Y-specific memory B cells detected after 3 priming doses (M7) compared to 2 priming doses (M6) (**p = 0.003 and p = 0.017 respectively; Fig 2). There was a decrease in the number of detectable memory B cells between the third priming dose (M7) and pre-boost (M12), although both antigens had a greater number of memory B cells pre-boost compared to post dose 2 of priming, and this was significant for Men Y (**p = 0.009). The largest detectable pool of memory B cells was present 1 month following a booster dose (M13) for both Men C and Men Y (as indicated by percentages below the x axis of each graph, Fig 2), where there was a significant increase in the number of Men C- (**p = 0.003) and Men Y- (**p = 0.001) specific memory B cells. ELISpot was also performed to detect Hib-specific responses, with detectable responses in 33.3% of children at M6, 26.1% at M7, 54.5% at M12 and 63.6% at M13; figure not shown).

### M12 Men C- specific memory B cell responses correlate with M13 SBA

**Fig 3:**
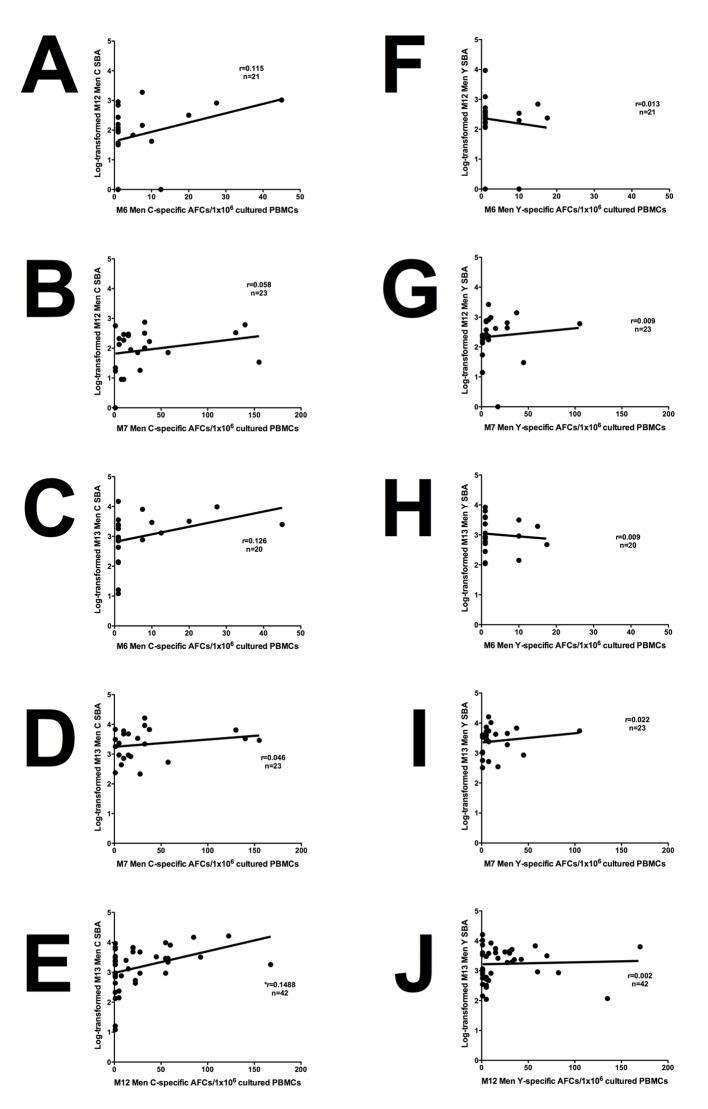
Association of Men C- and Men Y- specific memory B cells with SBA titre pre- and post- boost. Men C-specific memory B cells at M6 and M7 were plotted against log-transformed M12 Men C SBA (A and B) and M13 SBA (C and D). M12 Men C-specific memory B cells were plotted against log-transformed M13 SBA values (E). Men Y-specific memory B cells at M6 and M7 were plotted against log-transformed M12 Men Y SBA (F and G) and M13 SBA (H and I). M12 Men Y-specific memory B cells were plotted against log-transformed M13 SBA values (J). Linear regression analyses were performed to determine statistically significant associations where r = r^2^ value, n = number of subjects and p(*)≤0.05.

There was a significant positive association between M12 Men C-specific memory B cells and M13 Men C-specific SBA titre (Fig 3E; *r = 0.1488). Whilst there were positive associations between Men C-specific memory B cells and SBA titre at other time points, these were not significant (Fig 3A–3D). All associations between Men Y-specific memory B cells and SBA were not significant (Fig 3F–3J).

### TT-specific CD4+ T cell responses

**Fig 4:**
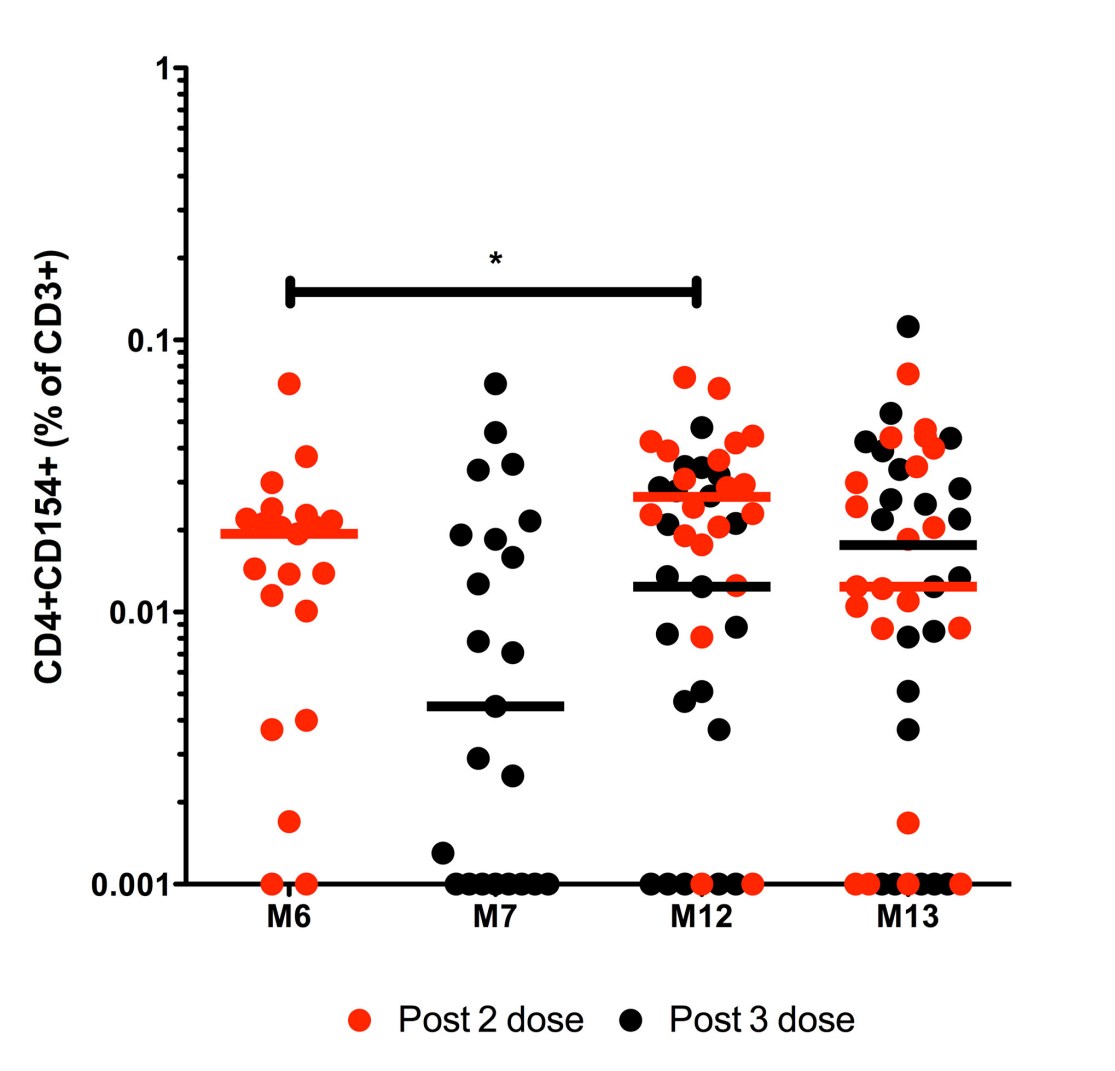
TT-specific CD4+CD154+ CD3+ responses over time following HibMenCY-TT vaccine. The frequency above background (Δ) of TT-specific CD4+CD154+ memory cells in CD3+ lymphocytes was determined in PBMCs from infants after 2 doses (M6) or 3 doses (M7) of prime, prior to (M12), and 1 month post (M13) a booster dose of HibMenCY-TT. Each point represents the response from 1 individual and the horizontal bars represent median values. Red dots represent paired samples from individuals who had blood taken following 2 doses of HibMenCY-TT prime, whilst black dots represent those samples from individuals who had blood taken after 3 doses of HibMenCY-TT prime. p(*)≤0.05, indicating significance of related samples Wilcoxon signed rank tests.

HibMenCY-TT vaccination induced TT-specific memory responses with median frequencies of 0.019% and 0.0045% CD4+CD154+ of CD3+ T cells after 2 or 3 priming doses respectively (Fig 4). There was a significantly greater frequency of TT-specific memory T cells pre-boost compared to post- 2 priming doses (median of 0.019% and 0.0265% at M6 and M12 respectively; p = 0.033). There were median frequencies of 0.021% and 0.0134% pre-boost and post-boost respectively (Fig 4).

### Total TT-specific CD4+ numbers do not correlate with Men C and Men Y SBA titres

**Fig 5:**
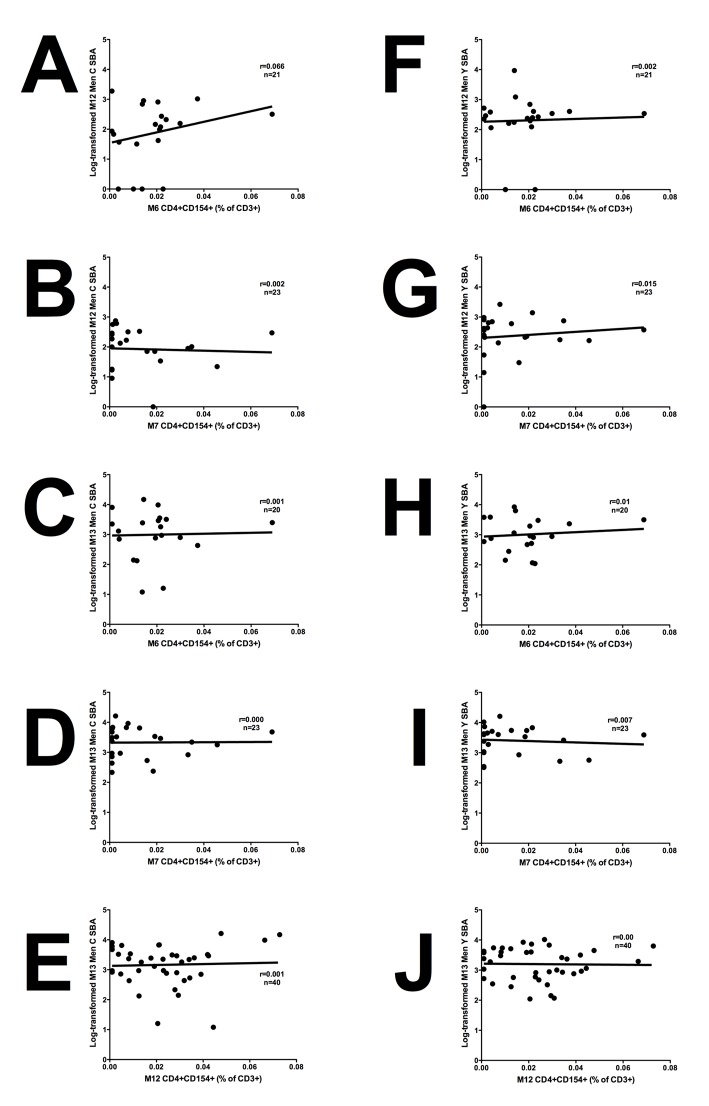
Association of TT-specific memory T cell responses with SBA titre pre- and post- boost. TT-specific CD4+CD154+ T cells at M6 and M7 were plotted against log-transformed M12 Men C SBA (A and B) and M13 SBA (C and D). M12 TT-specific CD4+CD154+ T cells were plotted against log-transformed M13 SBA values (E). TT-specific CD4+CD154+ T cells at M6 and M7 were plotted against log-transformed M12 Men Y SBA (F and G) and M13 SBA (H and I). M12 TT-specific CD4+CD154+ T cells were plotted against log-transformed M13 SBA values (J). Linear regression analyses were performed to determine statistically significant associations where r = r^2^ value and n = number of subjects.

The association between the number of TT-specific memory CD4+ T cells following priming (M6 and M7) and SBA titres pre- and post- boost (M12 and M13) were assessed to determine the influence of TT-specific memory CD4+ T cells on functional IgG persistence and booster response, but we found no significant associations (Fig 5A–5D, 5F–5I). The association between the frequency of TT-specific memory CD4+ T cells prior to boosting (M12) and SBA titre post boost was also assessed, but we found no significant associations (Fig 5E and 5J).

**Fig 6:**
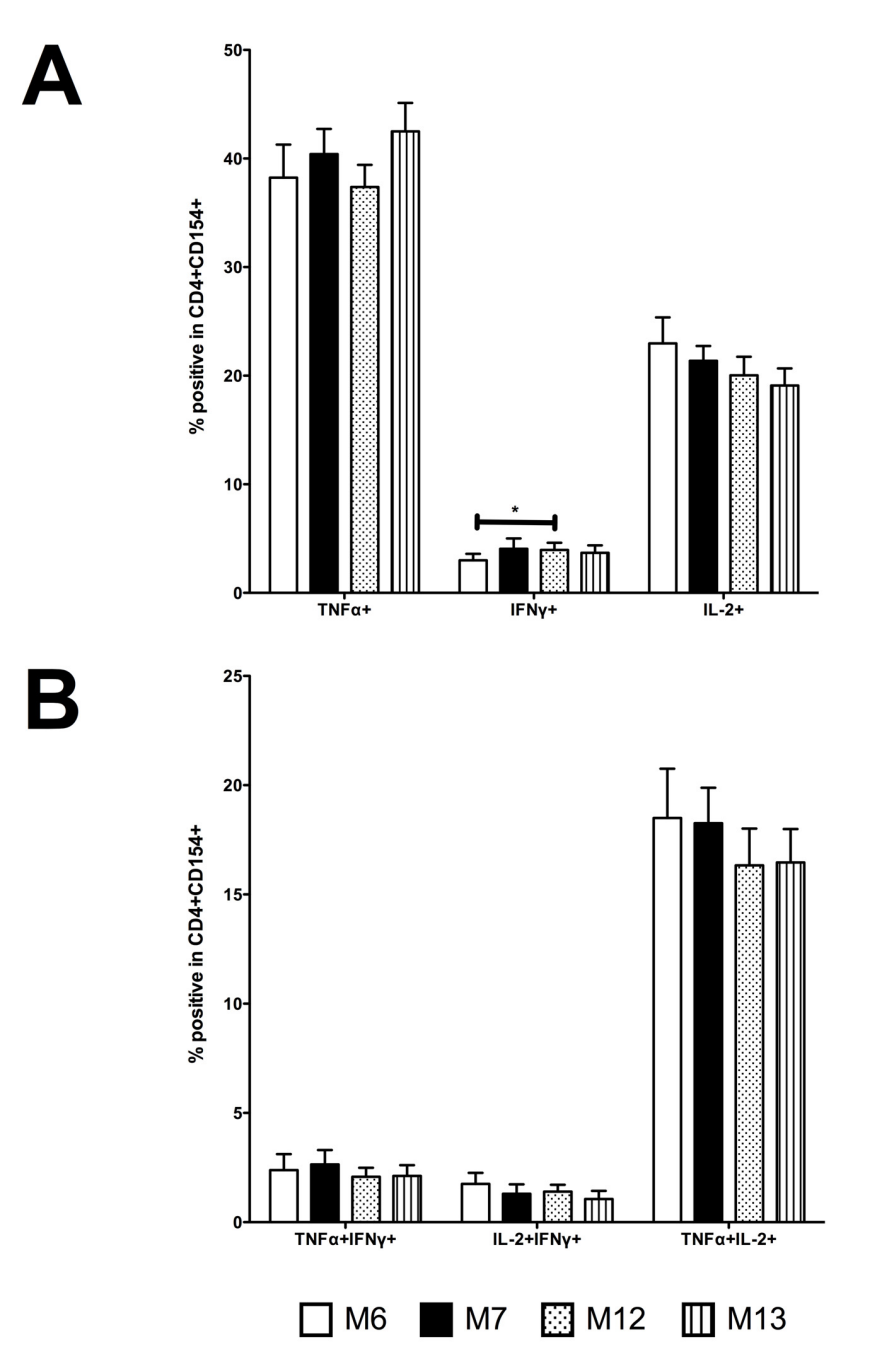
Single and double positive TT-specific memory CD4+ T cell cytokine responses following HibMenCY-TT vaccine. Intracellular TNFα, IFNγ, and IL-2 production by TT-specific memory cells was determined 48 hours after stimulation with TT, in PBMCs from infants after 2 doses (M6) or 3 doses (M7) of prime, and prior to (M12), and 1 month post (M13) a booster dose of HibMenCY-TT. **A.** single-positive TNFα+, IFNγ+ and IL-2+ cells and **B.** double-positive TNFα+IFNγ+, IL-2+IFNγ+ and TNFα+IL-2+ cells. Mean + SEM are shown. p(*)≤0.05 indicating significance of related samples Wilcoxon signed rank tests.

We measured the frequency of single and double positive cytokine expression within CD4+CD154+ T cells (Fig 6). To determine the effect of post-prime and pre-boost cytokine-producing TT-specific memory CD4+ T cells on SBA titre, we performed regression analyses. There were significant negative associations between M12 TNFα+ TT-specific memory CD4+ T cells and M13 Men C SBA (*r = 0.128, Table 2) and; M7 and M12 IFNγ+ TT-specific memory CD4+T cells and M13 Men Y SBA (*r = 0.178 and r = 0.123 respectively; Table 2). There was also a significant negative association between M12 TNFα+IL-2+ TT-specific memory CD4+ T cells and M13 Men C SBA (*r = 0.102; Table 3).

**Table 2:** Associations between single cytokine positive CD4+CD154+ T cells and Men C- and Y- specific SBA titre following regression analysis of non-parametric cytokine frequencies and log-transformed SBA values.

	M12 SBA		M13 SBA	
	Men C	Men Y	Men C	Men Y
**M6 TNFα+**	r = 0.018	r = 0.097	r = 0.119	r = 0.037
	n = 21	n = 21	n = 21	n = 21
**M7 TNFα+**	r = 0.009	r = 0.025	r = 0.026	r = 0.001
	n = 23	n = 23	n = 23	n = 23
**M12 TNFα+**			r = 0.128*	r = 0.073
			**n = 40**	n = 40
			*p = 0.024	
**M6 IFNγ+**	r = 0.011	r = 0.018	r = 0.049	r = 0.045
	n = 21	n = 21	n = 21	n = 20
**M7 IFNγ+**	r = 0.001	r = 0.077	r = 0.038	r = 0.178*
	n = 23	n = 23	n = 23	**n = 23**
				*p = 0.045
**M12 IFNγ+**			r = 0.033	r = 0.123*
			n = 40	**n = 40**
				*p = 0.027
**M6 IL-2+**	r = 0.052	r = 0.063	r = 0.118	r = 0.008
	n = 21	n = 21	n = 20	n = 20
**M7 IL-2+**	r = 0.000	r = 0.003	r = 0.006	r = 0.050
	n = 23	n = 23	n = 23	n = 23
**M12 IL-2+**			r = 0.043	r = 0.027
			n = 40	n = 40

r = r^2^ value, n = number of subjects using linear regression analyses.

**Table 3:** Associations between double cytokine positive CD4+CD154+ T cells and Men C- and Y- specific SBA titre following regression analysis of non-parametric cytokine frequencies and log-transformed SBA values.

	M12 SBA		M13 SBA	
	Men C	Men Y	Men C	Men Y
**M6 TNFα+IFNγ+**	r = 0.024	r = 0.028	r = 0.001	r = 0.051
	n = 21	n = 21	n = 20	n = 20
**M7 TNFα+IFNγ+**	r = 0.004	r = 0.019	r = 0.010	r = 0.052
	n = 23	n = 23	n = 23	n = 23
**M12 TNFα+IFNγ+**			r = 0.035	r = 0.045
			n = 40	n = 40
**M6 IL-2+IFNγ+**	r = 0.038	r = 0.059	r = 0.003	r = 0.022
	n = 21	n = 21	n = 20	n = 20
**M7 IL-2+IFNγ+**	r = 0.000	r = 0.040	r = 0.007	r = 0.107
	n = 23	n = 23	n = 23	n = 23
**M12 IL-2+IFNγ+**			r = 0.051	r = 0.031
			n = 40	n = 40
**M6 TNFα+IL-2+**	r = 0.038	r = 0.027	r = 0.132	r = 0.000
	n = 21	n = 21	n = 20	n = 20
**M7 TNFα+IL-2+**	r = 0.004	r = 0.000	r = 0.002	r = 0.016
	n = 23	n = 23	n = 23	n = 23
**M12 TNFα+IL-2+**			r = 0.102*	r = 0.026
			**n = 40**	n = 40
			*p = 0.04	

r = r^2^ value, n = number of subjects using linear regression analyses.

## Discussion

Immunogenicity studies have highlighted the need to understand the cellular determinants leading to persistent antibody responses following glyco-conjugate vaccination in infancy. In this study, we have detected memory B cells specific to Men C and Men Y, and CD4+ T cells specific to TT following a primary and booster course of HibMenCY-TT in infants. We have found that the number of Men C-specific memory B cells present pre-boost, is significantly associated with the post-boost SBA response; that the numbers of TT-specific CD4+ T cells post-priming or pre-boosting are not associated with Men C- and Men Y- specific IgG responses and; the frequency of TNF-α and IL-2 producing TT-specific CD4+ T cells pre-boost negatively correlates with polysaccharide-specific IgG post-boosting.

We detected memory B cells specific for Men C and Men Y in the peripheral blood from as early as 6 months of age, 2 months after 2 priming doses of vaccine. The significantly greater number of Men C- and Men Y- specific memory B cells at M7 compared to M6 is likely reflective of the time of blood sampling post vaccination, where M6 samples are 2 months following the previous dose of vaccine and M7 are 1 month post the previous dose of vaccine. Whilst a recent study comparing several Men C glyco-conjugate vaccines has shown an increase in the number of memory B cells from post-prime to pre-boost [12], we have not seen this in our study. This could be due to differences in the vaccines used, or the length of time between post-prime and pre-boost time-points. Overall, our findings are consistent with an earlier study of a Men C glyco-conjugate vaccine in which there was a decrease in the number of Men C-specific memory B cells between post-prime and pre-boost [7]. We found that the number of Men C- specific memory B cells at M12 were a predictor of the post-boost SBA titre, indicating that Men C-specific memory B cells present in the peripheral blood at M12 could be responsible for a significant component of the anamnestic functional IgG response following a HibMenCY-TT booster. These findings are in line with findings from studies of glyco-conjugates in which a polysaccharide challenge dose is given after priming, where higher IgG concentrations post-challenge indirectly indicate the differentiation of pre-formed memory B cells into antibody-forming plasma cells [18–20]. On a cellular level, this has previously been shown for Men C-specific responses following MenC-CRM_197_ when a fourth dose of MenC-CRM_197_ was given as a booster at 12 months of age [7]. The persistence of memory B cells in the circulation at 12 months is likely to indicate a transitory pattern in which they can re-enter germinal centres to form plasma cells following antigen-specific exposure, which is a hallmark of secondary responses [21–23]. These results are in contrast to a study of persistent memory B cells 6 years after primary vaccination of toddlers with Men C glyco-conjugate vaccine where there was no correlation with memory B cells at steady state and Men C-specific IgG responses 1 month later [10]. The length of time post-primary vaccination is shorter in our study however (6 months compared to 6 years), increasing the likelihood of these cells being available in the circulation for detection.

We did not see any relationship between the number of memory B cells formed at priming and pre-boost SBA titres. It has been suggested that whilst booster vaccination is able to restore IgG responses by driving antigen-specific differentiation of memory B cells, there is the potential for maintenance of persistent IgG prior to boosting through polyclonal stimulation of polysaccharide-specific memory B cells [23–25], and a similar study has shown an association between post-prime memory B cells and pre-boost IgG [7]. In contrast, the lack of association between the number of post-priming memory B cells and pre-boost SBA titre in our study suggests that primed memory B cells are highly dependent on antigen-specific signals for differentiation into plasma cells, discounting the contribution of polyclonal factors [25] or bacterial carriage [26] in maintenance of a polysaccharide-specific IgG response, at least in infants. Therefore, maintenance of serum IgG in the period between 6/7 months and 12 months is more likely to be due to plasma cells, previously highlighted by another study which addressed association between memory B cells and IgG in human peripheral blood [27]. The waning SBA titres in the period between priming and boosting is therefore most likely indicative of the inability of infants to support induction of long-lived plasma cells [28, 29].

In this study we have proposed that a greater number of TT-specific CD4+ T cells induced at priming would be a direct indicator of the persistence of functional polysaccharide-specific IgG, where their presence would increase both the quality of priming and the subsequent differentiation of memory B cells into plasma cells. To our knowledge, this is the first study to directly enumerate and phenotype the nature of the carrier protein-specific CD4+ T cell response following a glyco-conjugate vaccine, and to determine the influence of these cells on IgG responses. We found no association between TT-specific CD4+ T cells post-priming and pre-boost SBA, or between pre-boost TT-specific CD4+ T cells and post-boost SBA, suggesting what may be a threshold requirement of TT-specific CD4+ T cells at priming and a limited role for carrier protein-specific memory in maintenance of the Men C and Y specific IgG response. It is also possible that this lack of association between TT-specific CD4+ T cells and SBA is because TT-specific T cells are not in the peripheral blood at the time of detection, and rather in germinal centres in regional lymph nodes. Hence the lack of association is not necessarily indicative of what is happening *in vivo*. In contrast to our findings, a study of Pneumococcal conjugate vaccine in adults showed that CRM_197_ carrier protein-specific lympho-proliferative responses strongly correlate with the breadth of polysaccharide-specific IgG responses [30]. We expect that differences observed between our own study and this one could be due to a difference in the type of assay used to measure CRM_197_-specific responses, or the age of subjects, where the Rabian *et al* (2010) study was conducted in adults who would have been primed by pneumococcal carriage.

A lack of association between circulating TT-specific CD4+ T cells pre-boost and Men C- and Y- specific SBA post-boost suggests the recruitment of naïve CD4+ T cells in secondary germinal centre reactions. Indeed, a recent study in adults receiving MenC-TT vaccine has indicated a central role for bystander T cell activation in differentiation of polysaccharide-specific memory B cells into plasma cells [31]. In the context of our data, the presence of a greater number of primed TT-specific memory CD4+ T cells appears to be less relevant to down-stream IgG responses, once priming for B-cell memory has occurred, but this does not rule out the involvement of non-specific T cells in secondary responses.

To further investigate the role of TT-specific CD4+ T cells on polysaccharide-specific IgG responses, we measured the expression of key Th1 cytokines within the TT-specific cells, which strengthens the antigen-specific nature of these responses [32]. Although it was our intention to also investigate the nature of Th2 responses through IL-13 measurement, we were not successful in detecting a sufficient IL-13 signal. We saw no positive associations between cytokine-producing potential and SBA titre, but did see several significant negative associations between the presence of single positive TNF-α and IFN-γ expressing CD4+ T cells post-prime and pre-boost with SBA titre post-boost, and between the presence of double positive TNFα+IL-2+ expressing CD4+ T cells and Men C SBA titre post-boost. Despite the lack of association between TT-specific CD4+ T cells and IgG, there is a possibility that an environment in which Th1 cytokines are being produced in response to the carrier protein component of the conjugate is inhibitory to a strong polysaccharide-specific IgG response to booster. In mice, Jakobsen *et al* (2006) have shown that diminished IgG responses in neonates were a consequence of diminished TT-specific production of the Th2 cytokine IL-5 [33]. A potentially Th1-biased response prior to boosting in our study could support a similar phenomenon in human infants. However, previous research in adult humans and mice has also shown that there is independence in T cell cytokine responses and polysaccharide-specific IgG concentration following glyco-conjugate vaccination [34–36]. Our findings are in agreement with the latter studies, where we have seen no association between the number of TT-specific CD4+ T cells and functional IgG.

In conclusion, we have shown that Men C-specific memory B cells present pre-boost, presumably formed at glyco-conjugate vaccine priming, are influential in secondary IgG responses to HibMenCY-TT vaccine. The formation of primed carrier protein-specific CD4+ T cells appears to be independent of the IgG response, suggesting that with the help of sufficient numbers of CD4+ T cells at priming, B cells may intrinsically regulate their own fate. These findings have implications for the design of future glyco-conjugate vaccines, which should consider the requirements for promoting persistent IgG through control of B cell-regulated factors.
